# Unexpected maspin immunoreactivity in Merkel cell carcinoma

**DOI:** 10.1186/s13000-015-0437-3

**Published:** 2015-11-25

**Authors:** Sabin Gligore Turdean, Simona Gurzu, Ioan Jung, Radu Mircea Neagoe, Daniela Sala

**Affiliations:** Department of Pathology, University of Medicine and Pharmacy, 38 Ghe Marinescu Street, 540139 Tirgu-Mures, Romania; Department of Surgery, University of Medicine and Pharmacy of Tirgu-Mures, Tirgu-Mures, Romania

**Keywords:** Skin, Merkel cell carcinoma, Neuroendocrine, Maspin, DOG-1, TTF-1, bcl-2

## Abstract

Merkel cell carcinoma (MCC) is a rare but aggressive cutaneous neuroendocrine tumor, which multifactorial etiopathogenesis seems to be related to ultraviolet radiation, Merkel cell polyomavirus (MCV), and immunosuppression. In this paper, we present three cases of diagnosed MCC in apparently healthy Caucasians, two of them located in a sun-exposed area. They represented 0.25 % of all cutaneous malignant tumors diagnosed in our department. In the first case, MCC was diagnosed in the frontal region of a 67-year-old male, the second case was located in the right thigh of a 55-year-old female, whereas the third case involved the upper trunk of a 62-year-old female. All of these cases were diagnosed in the pT1 stage, having a diameter smaller than 2 cm, but the invasion depth involved the hypodermis. Microscopically, they consisted of small cells with round-oval nuclei having finely dispersed chromatin and well-defined nucleoli. Immunohistochemically, the tumor cells displayed positivity for keratin 20 and neuroendocrine markers, being negative for keratin 7 and S100 protein. Maspin immunoreactivity was seen in cases 1 and 3. Not one of the cases expressed DOG-1 or even TTF-1. Furthermore, this is the first report in literature about maspin positivity in MCC that might be related to sun exposure.

## Background

Merkel cell carcinoma (MCC) is a cutaneous neuroendocrine tumor firstly described by Toker Cyril in 1972 as trabecular carcinoma of sweat glands [1]. It mainly occurs on sun-exposed skin, especially in the head and neck area, followed by the extremities and trunk [2]. MCC is considered a rare but highly malignant tumor, being 40 times less common than malignant melanoma, with a 3-year mortality rate of 30 % and 27-61.5 % at 5-years, respectively, which is much lower compared to melanomas [2–5].

In a review published in June 2001, it was mentioned that only 836 cases of MCC have been reported in literature [6]. However, the incidence of MCC is significantly increasing, from 0.15 cases per 100,000 in 1986 to 0.44 in 2001 and to 0.6 cases per 100,000 in 2010 [7, 8]. The estimated annual percentage of change is about 8 % [7], with a tripling in the rate every 15 years [3]. MCC incidence in whites is 8 times higher than in blacks and almost double the incidence in other ethnic groups. MCC commonly affects men in all ethnic groups; the male:female ratio being about 1.5–2:1 [4, 9]. In the 9^th^ period of age, the incidence increases till 4.28 cases per 100,000 [7]. Compared with healthy people, the overall risk of MCC increases 5–23.8 times after solid organ transplantation [10] and 11 times in patients with AIDS, respectively [11]. However, even in the large diagnostic centers, only 3–5 cases of MCCs per year are diagnosed, and most of these cases are published as case reports. Early diagnosis is important, a routine skin screening being recently proposed for non-melanoma skin cancers [12].

Although ultraviolet radiation, immunosuppression, and Merkel cell polyomavirus (MCV) infection are considered the main factors responsible for carcinogenesis of MCC [5, 13], the molecular mechanism is poorly understood, and the neuroendocrine arhitecture makes the differential diagnosis very difficult. In this paper, we report the pathologic findings, criteria for differential diagnosis, and the particularities of the immunoprofile of MCCs, based on three cases and a comprehensive literature review. A hypothesis about the possible role of maspin in carcinogenesis of MCC was also postulated first time in literature. The disease stage was evaluated based on the 2010 AJCC TNM classification [14]. In all of the cases, a signed informed consent of the patients was obtained for performing surgery and publication of the case-related data.

## Case presentation

To select the cases, a database of 3,410 consecutive cutaneous tumors was evaluated. From these, 1,196 were malignant tumors, and the other 2,214 being benign tumors or pseudotumors. Only 3 out of 1,196 cutaneous malignant tumors were diagnosed as MCCs (0.25 %).

### Case 1

**Fig. 1:**
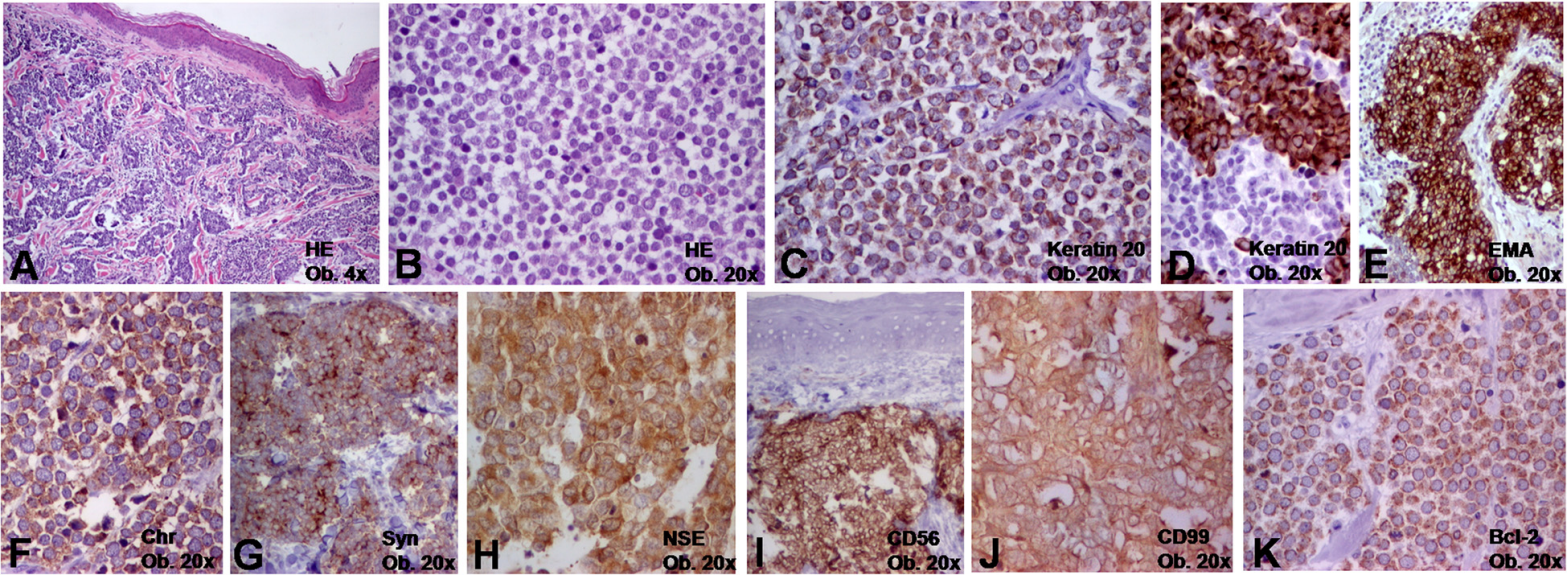
Microscopically, the Merkel cell carcinoma is characterized by intradermal proliferation of clusters of small cells (**a**, **b**) that are marked by keratin 20 (**c**, **d**), epithelial membrane antigen (**e**), the neuroendocrine markers (**f**-**i**), CD99 (**j**), and bcl-2 (**k**) (Chr = chromogranin; EMA = epithelial membrane antigen; NSE = neuron specific enolase; Syn = synaptophysin)

**Fig. 2:**
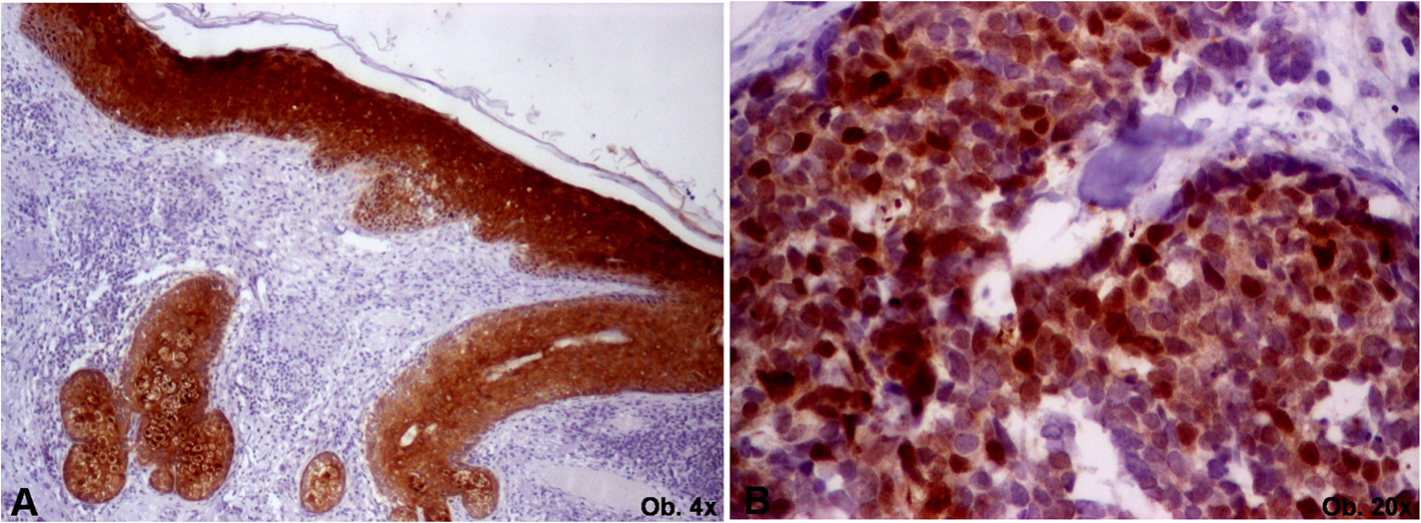
Microscopically, maspin cytoplasmic positivity can be seen in normal epithelium (**a**) whereas Merkel cell carcinoma cells express nuclear expression (**b**)

A 67-year-old previously healthy white male was hospitalized with a slowly growing 17×17×8 mm nodular ulcerated tumor located on the frontal region that was surgically removed, with free resection margins. Microscopically, the tumor consisted of nests of small round cells with scanty cytoplasm and round-oval nuclei with finely dispersed chromatin and well defined nucleoli (Fig. 1). Nuclear pleomorphism was moderate with a high mitotic rate (>10/10 HPF). The tumor cells infiltrated the whole dermis and subcutaneous adipose tissue, and the maximum thickness was of 8 mm. Based on the tumor size, histological aspect, immunoprofile (Table 1), and absence of lymph node metastases, the tumor was diagnosed as pT1N0-stage MCC. Nuclear maspin positivity (Fig. 2) was unexpected.

**Table 1:** Clinicopathological features of Merkel cell carcinoma

Case no	Age (years)	Location	Sex	Size (mm)	Ulceration	Stage	Depth of invasion	Mitotic rate (per 10 HPF)	Positive IHC markers	Negative IHC markers
1	67	Frontal region	Male	17×17×8	Present	pT1	Subcutaneous adipose tissue	30	AE1/AE3 keratin, keratin 20, EMA, Chromogranin, Synaptophysin, NSE, CD56, CD99, bcl-2, maspin	Vimentin, Keratin 7, S-100 protein, desmin, CEA, CD20, CD3, DOG-1, HMB45, E-cadherin, CD3, CD20, CD10, TTF-1
2	55	Right thigh	Female	10×10×5	Absent	pT1	Subcutaneous adipose tissue	15	keratin20, Chromogranin, Synaptophysin, NSE, CD99, bcl-2	Vimentin, Keratin 7, S-100 protein, desmin, CEA, CD20, CD3, DOG-1, HMB45, E-cadherin, CD3, CD20, CD10, TTF-1, maspin
3	63	Thoracic region	Female	12×12×12	Absent	pT1	Subcutaneous adipose tissue	3	AE1/AE3 keratin, keratin 20, EMA, Chromogranin, Synaptophysin, NSE, CD56, CD99, bcl-2, maspin	Vimentin, Keratin 7, S-100 protein, desmin, CEA, CD20, CD3, DOG-1, HMB45, E-cadherin, CD3, CD20, CD10, TTF-1

*Abbreviations: CD* cluster of differentiation, *CEA* carcinoembryonic antigen, *EMA* epithelial membrane antigen, *IHC* immunohistochemistry, *NSE* neuron specific enolase; Maspin-mammary serine protease inhibitor, *TTF* thyroid transcription factor

With any postoperative therapy, the patient is still alive without any recurrences or metastases at two years of follow-up.

### Case 2

A 55-year-old previously healthy white female presented a 10×10×5 mm nodular, non-ulcerated skin tumor located on the right thigh. Surgical excision was performed. The microscopical aspect was similar to those described in Case 1, but the nuclear pleomorphism was moderate, the mitotic rate was of 8 mitoses/10 HPF, and tumor cells were negative for Maspin. The whole dermis and subcutaneous adipose tissue were involved, the maximum thickness was 5 mm, and all of the resection margins were infiltrated. The histological aspect and immunoprofile suggested a primary MCC (Table 1). The final diagnosis was pT1-staged MCC. No lymph nodes were excised. The patient did not come back for further therapy and is alive at one month after surgery.

### Case 3

A 62-year-old female presented a 12×12×12 mm nodular non-ulcerated tumor of the upper trunk (sun-exposed area) that was surgically removed. The tumor nests displayed the same microscopically features as in the other two cases, the dermis and subcutaneous adipose tissue was infiltrated and the maximum thickness was of 12 mm. Minimal pleomorphism and <3 mitoses/10 HPF were noted. Because the deep and lateral resection margins were infiltrated by the tumor cells, a re-excision was necessary. The final diagnosis, after re-excision, was pT1-stage MCC that was confirmed by the tumor cells immunoprofile (Table 1). The margins were found to be microscopically uninvolved by carcinoma. Distance of carcinoma from closest margin: 2/2/2 mm (Peripheral Margins and Deep Margin). Unusual nuclear maspin positivity was observed in the tumor cells. No lymph nodes were excised and no recurrences or metastases were reported six months after surgery. In addition, no radiotherapy was performed.

In all three cases there was no association with conditions indicating impaired immune status (organ transplantation, including renal, cardiac, as well as bone marrow, receiving immunosuppressive therapy for rheumatoid arthritis and with aplastic anemia or lymphoma, HIV infection, chronic lymphocytic leukemia, arsenic ingestion, tumor after radiation therapy).

## Discussion

MCC preponderantly produces early metastases in regional and/or distant lymph nodes. Most of the cases (53 %) are diagnosed in stage III with metastases in more than 4 lymph nodes, and only 13 % of them being identified in stage I [3], such in our cases. About 20–30 % of MCCs are diagnosed with distant metastases [15, 16]. The 5-year survival rate depends on the tumor size, ranging from 66–75 % in tumors smaller than 2 cm to 50–60 % in those larger than 2 cm. Positive margins, absence of postoperative radio-chemotherapy, patient’s age (older than 75 years), relapses, and metastases are also considered important prognostic factors [3, 15–17]. The 5-year survival rate decreases from 42–52 % in node-positive MCCs to 17–18 % in cases with distant metastases [18]. In every non-metastatic case (stages I and II), wide excision with safety limits and sentinel lymph node biopsy is recommended, followed by radiotherapy [13]. In node-positive MCCs (stage III), treatment of the nodal basin with lymphadenectomy and radiotherapy should be performed [16]. In cases with distant metastases (stage IV), platinum-based chemotherapy and/or immunotherapy should be associated [16]. Oblimersem sodium can be used in bcl-2 positive cases or lorvotuzamab mertansine in CD56-positive MCCs, but the results are not very well known [16]. The newest drug proposed to be used for patients with metastatic MCC is the PI3K pathway inhibitor called Idelalisib that was recently approved by the Food and Drug Administration for application in B-cell lymphoma [19].

Diagnosis of MCC is very difficult and should be based on the clinico-pathological parameters such as tumor location, patient’s age (being more frequent in sun-exposed areas and older people) correlated with the histological neuroendocrine aspect, and absence of the contact of tumor cells with the epidermis. In few cases epidermotropism and additional divergent components such as squamous, follicular, porocarcinoma, sarcomatous, glandular, and neuroblastic were noted [5]. However, the final diagnosis depends on the tumor profile and should take into account a metastasis from tumors with round cells (small cell lung carcinoma, neuroendocrine carcinoma of other organs, neuroblastoma), other cutaneous carcinomas with round cells (sweat gland carcinoma, basal cell carcinoma with neuroendocrine differentiation, small cell squamous cell carcinoma, primary cutaneous small cell carcinoma), lymphomas, and melanomas.

The immunohistochemical characteristics expression of neuroendocrine markers correlated with perinuclear dot-like positivity of keratin 20 is considered specific for MCC [4, 20]. However, unusual immunopositivity of the tumor cells was also reported (Table 2). The monoclonal antibody CM2B4 marker was also introduced in 2009 in clinical practice, which acts against a predicted antigenic epitope on the MCV T-antigen and can be added in the daily diagnosis panel of antibodies [20, 21]. In some of the cases, keratin 20 can be negative, especially in carcinomas unrelated with MCV infection [22]. Unusual positivity was reported for markers such as TTF-1 [23], CD57, PAX-5, TDT (terminal deoxynucleotidyl transferase) [24], and maspin, first reported in this study.

**Table 2:** Differential diagnosis of Merkel cell carcinoma based on the tumor cells immunoprofile [2–34]

IHC marker	MCC	BCC with neuroendocrine differentiation	Melanoma	SCLC - skin metastasis
AE1/AE3 Keratin	+	+	±	+
Keratin 20	±	-	-	-
Keratin 7	±	-	-	+
EMA	+	+	-	+
CEA	-	-	-	-
Chromogranin	±	±	-	±
Synaptophysin	+	±	-	±
NSE	±	±	-	±
CD56	±	±	-	+
CD99	+	-	-	-
Bcl-2	±	-	-	-
S-100 protein	+	-	+	-
TTF-1	±	-	-	+
Vimentin	-	-	+	-
TDT	±	-	-	±
DOG-1	-	±	-	-
HMB45	-	-	±	-
Melan A	-	-	+	-
E-cadherin	-	+	-	-
Maspin	±	±	±	±
CD57	±	-	±	±
PAX-5	±	-	-	-
c-KIT	±	-	±	-

Abbreviations: *BCC* basal cell carcinoma, *CD* cluster of differentiation, *CEA* carcinoembryonic antigen, *EMA* epithelial membrane antigen, *IHC* immunohistochemistry, *MCC* Merkel cell carcinoma, *NSE* neuron specific enolase; Maspin-mammary serine protease inhibitor, *SCLC*-small cell lung carcinoma, *TDT* terminal deoxynucleotidyl transferase, *TTF* thyroid transcription factor

Differentiation of MCC from cutaneous metastases of neuroendocrine carcinoma is difficult and can be based on CEA negativity (that is usually positive in tumors of the gastrointestinal tract and pancreas) and inconstant positivity for keratin 20 [5, 6]. Predominantly, TTF-1 negativity and PAX-5 positivity of MCC is a diagnostic tool in differentiation from a metastatic lung cancer, although inconstant positivity was also observed in MCC [16, 23, 24]. Regarding the primary cutaneous small cell carcinoma, this lesion is characterized by the complete absence of nucleoli, which are well visible in MCC, and keratin 20 negativity [25]. Moreover, the primary cutaneous small cell carcinomas including basal cell carcinoma with neuroendocrine differentiation are negative for keratin 20 [20, 26].

The first description of the Merkel cells was performed in 1875 by Friedrich Sigmund Merkel, who called them “tastzellen” or “touch cell” [27]. Further studies proved, using the electron microscope, that they are located in the basal layer of epidermis and dermis and play a role on slowly adapting mechanoreceptors to sense touch and hair movement [9, 16]. Merkel cells display a monomorphic aspect with scanty cytoplasm and nuclei with fine chromatin; the MCC showing proliferation of similar cells with nuclear pleomorphism.

The role of MCV infection was also recognized in 2008 as a predisposing factor for genesis of MCC. However, due to the fact that MCC can arise in the background of chronic radiodermatitis in patients negative for MCV [28] and mostly occurs in the sun-exposed areas, supposition that ultraviolets can induce activation, proliferation, and malignization of some pluripotent stem cells was evolved [16]. Also, some studies have shown that MCC with divergent differentiation is an aggressive subtype, in whose development MCV is not involved [4, 5].

In this paper, unusual nuclear maspin maspin positivity was noted in both of the cases that occured in the sun exposed areas (case 1 - face and case 3 - upper trunk), without any correlation with the mitotic rate, depth of infiltration, or the quality of the resection margins. The normal epidermis showed a cytoplasmic positivity. However, being about first report in literature about maspin expression in MCC, it is difficult to emit suppositions about its role in this cutaneous tumor, further studies being necessary to confirm its positivity in larger cohorts.

Maspin (mammary serine protease inhibitor) is a member of the serine protease inhibitor family that is knows to play a tumor suppressor role in several malignant epithelial tumors such as colorectal or gastric carcinomas [29–31]. However, its prognostic role depends on the subcellular localization, the p53-mediated nuclear positivity usually indicating a more aggressive behavior, a higher risk for tumor relapse and lymph node metastases, whereas loss of expression proved to induce a higher risk for distant metastases, at least for gastrointestinal malignant tumors [30, 31].

In tumors of the skin, maspin immunoreactivity was described in 97 % of squamous cell carcinomas and 88 % of basal cell carcinomas, but also in malignant melanomas, more frequent in sun-exposed areas [32, 33]. Few than 25 papers regarding maspin expression in cutaneous tumors have been published to date. However, there are limited data regarding correlation of maspin immunoreactivity with the prognosis. In squamous cell carcinomas, maspin positivity rate was higher in early stages compared with the advanced staged tumors (61 % vs. 39 %) and in non-metastatic tumors compared with cases that displayed lymph node positivity (67 % vs. 33 %). In bout squamous- and basal cell carcinomas it was especially displayed by tumors of the head and neck area (70 % vs. 30 %) [34]. A possible sun-activated maspin-induced DNA damage was also supposed [33]. On the other hand, nuclear expression proved to have a tumor suppressor role in basal cell carcinoma [34] but indicated poorer survival in melanomas [33].

The supposition about role of maspin in carcinogenesis of non-melanoma skin tumors that include MCC should be tested in further studies, on large cohorts.

## Conclusion

This report shows that Maspin positivity in Merkel cell carcinoma might be related on sun exposure.

## Consent

Written informed consent was obtained from the patients for publication of this Case Report and any accompanying images. A copy of the written consent is available for review by the Editor-in-Chief of this journal.
